# Bradycardia as a Rare Sign of Pulmonary Embolism: A Case Report of Pulmonary Embolism Diagnosis in Cardiac Arrest Using Point-of-Care Ultrasound

**DOI:** 10.7759/cureus.32798

**Published:** 2022-12-21

**Authors:** Muhammad Awais Cheema, Salman Naeem, Amir Alzarrad, Tony Joy, Haythem Jarad

**Affiliations:** 1 Emergency Department, Worthing Hospital, University Hospitals Sussex NHS Foundation Trust, Worthing, GBR; 2 Emergency Department, Barts Health NHS Trust, London, GBR

**Keywords:** cardiac arrest outcome, point-of-care-ultrasound, pulmonary embolism, emergency medicine resuscitation, emergency medicine, drugs and medicine, respiratory system, venous thromboembolism < cardiovascular medicine

## Abstract

We describe a case of cardiac arrest with pulmonary embolism and deep venous thrombosis diagnosed by point-of-care ultrasound, which resulted in a favorable outcome. In this article, we have also delineated bradycardia as an atypical sign of pulmonary embolism and explained the potential mechanism behind it.

## Introduction

Acute pulmonary embolism (PE) can be a life-threatening disorder with mortality as high as 30% in untreated cases [1]. With an annual incidence of around 60-70 cases per 100,000 people, it is one of the common emergencies presenting to the emergency department (ED) [2]. Patients usually present with tachycardia, dyspnea, cough, hemoptysis, and chest pain. Dyspnea is reported by more than 75% of patients; however, PE can also present as syncope, abdominal pain, and seizures [3]. Wells criterion for PE [4] is a validated tool to determine the estimated pre-test probability of PE, but the diagnosis relies heavily on the clinical gestalt of the physician. As a result of variability in the clinical presentation, a PE diagnosis in the ED can be challenging. This is a case report of an atypical presentation of PE in a patient who presented with syncope and bradycardia.

## Case presentation

A 48-year-old woman with a history of end-stage renal disease (ESRD), epilepsy, and transplant nephrectomy experienced a sudden collapse while awaiting her hemodialysis session. She was mildly hypoxemic but otherwise hemodynamically stable (heart rate: 76, blood pressure: 111/74 mmHg, oxygen saturation: 90% on room air, temperature: 35.6°C, and Glasgow Coma Scale [GCS]: 15/15). She was transferred to ED for further management. Approximately, one-minute post-arrival, she became unresponsive, with a heart rate of 20 bpm. The patient demonstrated jugular vein distension (JVD). She then became pulseless and went into cardiac arrest. Cardiopulmonary resuscitation was commenced immediately.

**Figure 1:**
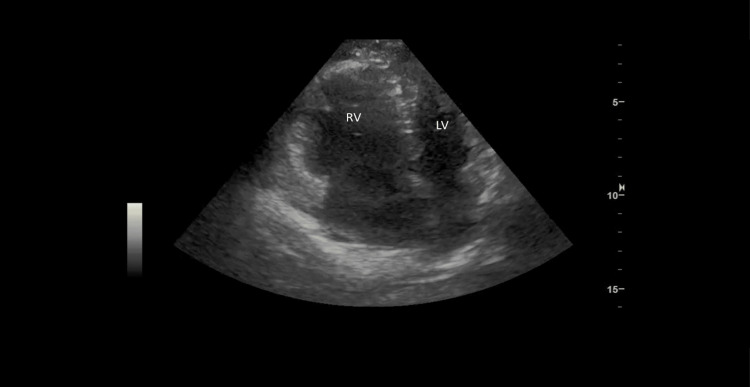
Apical four-chamber view of the heart showing massively dilated right ventricle (RV) as compared to the left ventricle (LV)

Intra-arrest bedside ultrasound examination was performed, which showed a dilated right ventricle (RV) (Figure 1), a septal bulge toward the left ventricle, and a hypertrophic left ventricle with a severely reduced ejection fraction.

**Figure 2:**
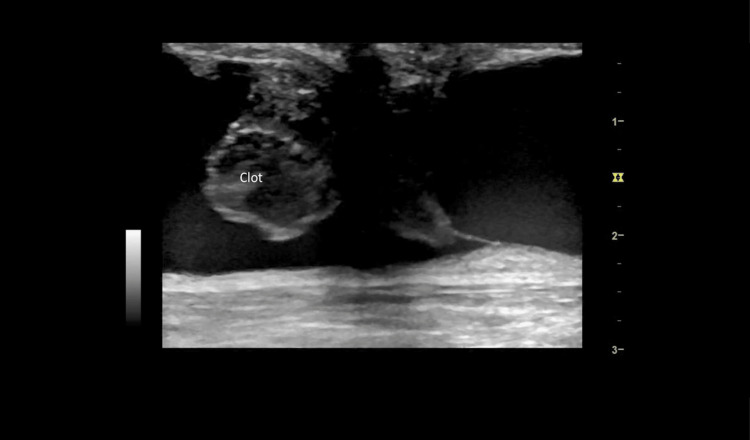
Linear view of the right brachiobasilic fistula with hyperechoic masses, likely clots, hanging by fibrinous band

**Figure 3:**
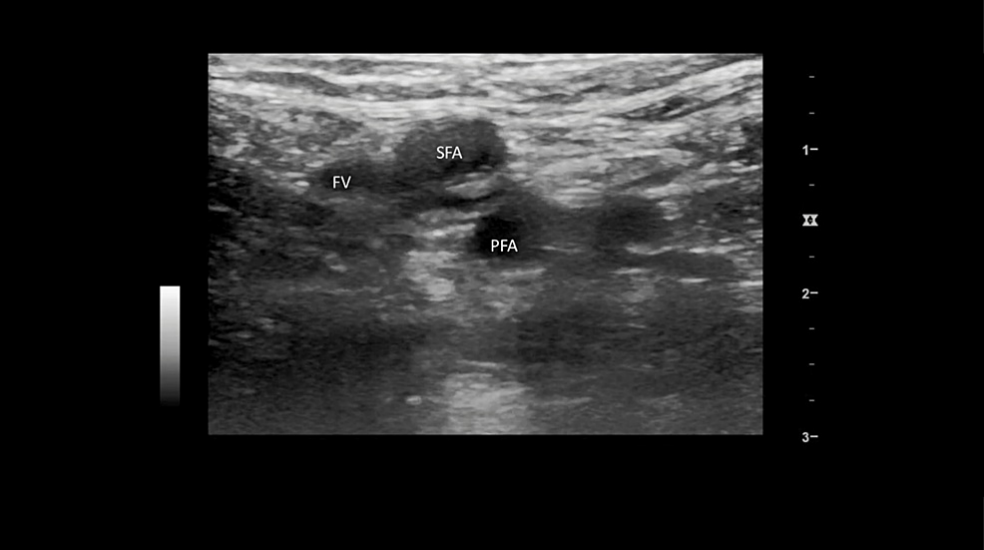
Transverse view of the superficial tissue of the left inguinal area with compression, showing the left superficial femoral artery (SFA), left profunda femoris artery (PFA) underneath, and noncompressible left femoral vein (FV) left lateral to SFA and PFA

The scan also showed clots in the right brachiobasilic arteriovenous fistula (BB-AVF) (Figure 2) and a deep vein thrombosis (DVT) in the left common femoral vein (Figure 3). The patient was consequently thrombolyzed with a 50-mg intravenous bolus of alteplase. This was followed by an intravenous infusion of 50 mg alteplase given over 30 minutes. She also received 5000 units of heparin.

**Figure 4:**
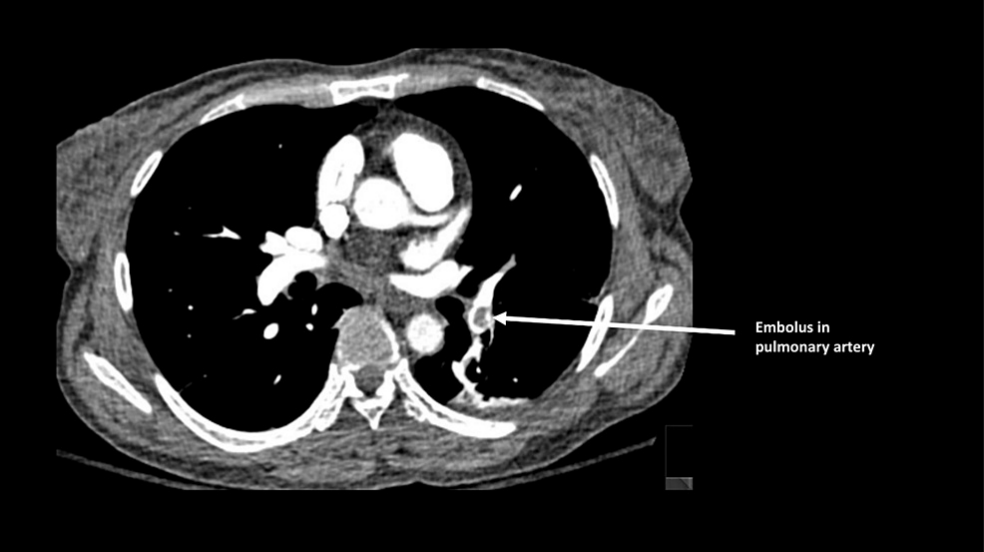
Axial view of computerized tomographic (CT) pulmonary angiogram showing a radiolucent clot in the left pulmonary artery depicted by the arrow

She had a return of spontaneous circulation and underwent a computerized tomographic (CT) pulmonary angiogram and CT of the abdomen and pelvis, which confirmed bilateral PE (Figure 4) and clots in both common femoral veins. She was admitted to the intensive care unit (ICU) where she remained stable. She was discharged on anticoagulation on hospital day 3 with no neurological deficit with further follow-up in the outpatient hematology clinic.

## Discussion

This case report highlights an atypical manifestation of a massive PE. In this patient, syncope and sinus bradycardia were two anomalous signs which are not commonly attributed to acute PE. The fact that the patient was arrested soon after her admission to ED limited the history and examination. The obstructive shock was clinically first suspected after noticing that the patient had distended jugular veins. Jugular venous pressure (JVP) can be raised due to various reasons including an increase in right heart pressure, volume, or occlusion of the superior vena cava (SVC). In acute PE, blood flow through the pulmonary arteries is obstructed. Pulmonary artery occlusion causes elevated right ventricular pressure through several mechanisms [5]. The acute rise in right ventricular pressure with resultant right heart failure in a massive PE is reflected by a raised JVP.

Although PE has long been recognized as an important and serious cause of syncope, its prevalence in patients presenting to the ED with syncope is actually rare, with a rate of diagnosis ranging from 0.06% to 0.55% [6]. It is proposed that PE causes syncope via three different mechanisms: (i) massive PE causing reduction in cardiac output leading to decreased perfusion of the cerebrum, (ii) a complete heart block in pre-existing left bundle branch block, and (iii) stimulation of vagal mechanosensory nerve endings in the left ventricle leading to cardiogenic shock and bradycardia [7]. All of these mechanisms reduce cardiac output that limits cerebral perfusion, causing loss of consciousness and syncope.

Contrary to the common clinical perception of acute PE being associated with persistent tachycardia, large cohort clinical studies have shown that sinus bradycardia may be present in more than 2% of patients with PE [8]. This bradycardia may be a result of a complete atrioventricular (AV) block due to an injury to the right bundle branch that runs superficially in the RV wall and in the intraventricular septum and is sensitive to acute RV dilatation in a patient with pre-existing electrical conduction system disease like left bundle branch block (LBBB) [5]. Alternatively, bradycardia can occur due to RV dilation and pressure overload from PE leading to excess vagal stimulation [9]. In this case, the patient first had an episode of syncope and then developed profound sinus bradycardia just before losing cardiac output. It is postulated that extracellular ATP could play a mechanistic role in syncope and bradycardia associated with PE. The mechanisms suggested are localized release of ATP by platelet activation and ATP-induced pulmonary-pulmonary and cardio-cardiac vagal stimulation [7]. The excess vagal stimulation causes negative chronotropic and dromotropic effects on the heart. This phenomenon along with mechanical obstruction in blood flow can lead to profoundly reduced cardiac output, resulting in syncope and subsequent cardiac arrest.

Patients with ESRD receiving hemodialysis are more at risk of having venous thromboembolism. Königsbrügge et al. found that the incidence of venous thromboembolism (VTE) is 10.9/1000 patient-years in ESRD patients [10] as compared to 0.9/1000 patient-years in the normal population [11]. Bedside echo has been shown to aid in the diagnosis of PE. The typical signs on echo are increased RV to left ventricle ratio, McConnell’s sign, tricuspid regurgitation, decreased tricuspid annular plane excursion, and D-sign [12]. Although these signs are non-specific, they help to raise the clinical suspicion of PE in a patient with high pre-test probability. Most PEs originate from a lower limb [13]. Compression ultrasonography (CUS) for DVT has a sensitivity of 90% and a specificity of 95% [14]. Right heart strain on echo and positive CUS in the lower limbs of DVT in a high-risk hemodynamically unstable patient warrants systemic thrombolysis according to the European Society of Cardiology guidelines for pulmonary embolism [15]. On subsequent sonography of this patient's upper limb BB-fistula, further blood clots were visualized.

## Conclusions

Point-of-care ultrasound in cardiac arrest can aid in the early diagnosis of the cause of cardiac arrest, which might improve the patient's outcome. In high-risk patients receiving hemodialysis, PE should be considered a differential diagnosis of syncope. In addition, bradycardia can be a confounding sign in the presentation of acute PE.

## References

[1] Bĕlohlávek J, Dytrych V, Linhart A (2013). Pulmonary embolism, part I: epidemiology, risk factors and risk stratification, pathophysiology, clinical presentation, diagnosis and nonthrombotic pulmonary embolism. Exp Clin Cardiol.

[2] Oger E (2000). Incidence of venous thromboembolism: a community-based study in Western France. EPI-GETBP Study Group. Groupe d'Etude de la Thrombose de Bretagne Occidentale. Thromb Haemost.

[3] Altınsoy B, Erboy F, Tanrıverdi H, Uygur F, Örnek T, Atalay F, Tor M (2016). Syncope as a presentation of acute pulmonary embolism. Ther Clin Risk Manag.

[4] Wells PS, Anderson DR, Rodger M (2001). Excluding pulmonary embolism at the bedside without diagnostic imaging: management of patients with suspected pulmonary embolism presenting to the emergency department by using a simple clinical model and d-dimer. Ann Intern Med.

[5] Bryce YC, Perez-Johnston R, Bryce EB, Homayoon B, Santos-Martin EG (2019). Pathophysiology of right ventricular failure in acute pulmonary embolism and chronic thromboembolic pulmonary hypertension: a pictorial essay for the interventional radiologist. Insights Imaging.

[6] Costantino G, Ruwald MH, Quinn J (2018). Prevalence of pulmonary embolism in patients with syncope. JAMA Intern Med.

[7] Pelleg A, Schulman ES, Barnes PJ (2018). Adenosine 5'-triphosphate's role in bradycardia and syncope associated with pulmonary embolism. Respir Res.

[8] Stein PD, Matta F, Sabra MJ (2013). Relation of electrocardiographic changes in pulmonary embolism to right ventricular enlargement. Am J Cardiol.

[9] Majidi A, Mahmoodi S, Baratloo A, Mirhaba S (2014). Atypical presentation of massive pulmonary embolism, a case report. Emerg (Tehran).

[10] Königsbrügge O, Lorenz M, Auinger M (2017). Venous thromboembolism and vascular access thrombosis in patients with end-stage renal disease on maintenance hemodialysis: cross-sectional results of the Vienna InVestigation of AtriaL fibrillation and thromboembolism in patients on hemoDIalysis (VIVALDI). Thromb Res.

[11] Tagalakis V, Patenaude V, Kahn SR, Suissa S (2013). Incidence of and mortality from venous thromboembolism in a real-world population: the Q-VTE study cohort. Am J Med.

[12] Alerhand S, Sundaram T, Gottlieb M (2021). What are the echocardiographic findings of acute right ventricular strain that suggest pulmonary embolism?. Anaesth Crit Care Pain Med.

[13] Hull RD, Hirsh J, Carter CJ (1983). Pulmonary angiography, ventilation lung scanning, and venography for clinically suspected pulmonary embolism with abnormal perfusion lung scan. Ann Intern Med.

[14] Kearon C, Ginsberg JS, Hirsh J (1998). The role of venous ultrasonography in the diagnosis of suspected deep venous thrombosis and pulmonary embolism. Ann Intern Med.

[15] Konstantinides SV, Meyer G, Becattini C (2020). 2019 ESC Guidelines for the diagnosis and management of acute pulmonary embolism developed in collaboration with the European Respiratory Society (ERS). Eur Heart J.

